# Is there a causal association between temporomandibular disorders and COVID-19 risk? A genetic instrumental variables analysis

**DOI:** 10.22514/jofph.2024.018

**Published:** 2024-06-12

**Authors:** Jiayi Chen

**Affiliations:** Department of Stomatology, Suzhou Wujiang District Hospital of Traditional Chinese Medicine, 215221 Suzhou, Jiangsu, China

**Keywords:** COVID-19, Temporomandibular joint disorders, Mendelian randomization analysis, Psychosocial factors, Biostatistics

## Abstract

The coronavirus disease 2019 (COVID-19) and temporomandibular joint disorders (TMD) as the two major diseases are being focused by the public in modern societies. Previous epidemiological studies have shown increase in TMD prevalence during COVID-19 pandemic era. This study was aimed to verify the causal association between two sides using bidirectional mendelian randomization (MR) analysis. It explored whether COVID-19 could cause TMD or TMD influenced the COVID-19 susceptibility. Furthermore it was aimed to eliminate the reverse relationship and other confounders, and an attempt was made to provide etiologic evidence. Single-nucleotide polymorphisms (SNPs) related to three COVID-19 phenotypes (*p *< 5 × 10^−8^) were selected from the genome-wide association study (GWAS) data collected through COVID-19 host genetics initiative (HGI). SNPs related to TMD (*p *< 5 × 10^−6^) were collected from GWAS data in UK Biobank (UKB). Inverse variance weighted (IVW), weighted median (WM), and MR-Egger regression estimated the causal effect between two sides in this study. Furthermore, four sensitivity analyses (MR-PRESSO, Cochran’s Q test, MR-Egger intercept test, and leave-one-out test) were used to confirm the robust results. TMD-related GWAS in FinnGen repeated the MR to validate the results. COVID-19 was not affected by TMD. The reversed MR suggested no significant causal effect of COVID-19 on TMD. Sensitivity analyses showed no gene pleiotropy and had robust results in this MR. Nonetheless, the MR statistical power was <80%, which suggested insufficient sample size of COVID-19 and TMD. This study based on current evidence depicted that COVID-19 had no impact on TMD, and TMD did not increase the susceptibility of severe acute respiratory syndrome coronavirus-2. During COVID-19 pandemic, excessive psychological stress caused by COVID-19 might act as a mediator between the two diseases. The relationship between the two sides needs verification by more external studies in the future.

## 1. Introduction

COVID-19 pandemic caused by severe acute respiratory syndrome coronavirus-2 has threatened the public health worldwide [1]. Severe acute respiratory syndrome coronavirus-2 is like a bat coronavirus at whole-genome level which can be classified as the B lineage of β-coronaviruses [2]. Chinese experts reported the most common symptoms of COVID-19 as fever (43.8% on admission and 88.7% during hospitalization), cough (67.8%), and diarrhea (3.8%) [3]. COVID-19 pandemic had adverse impact on society, economy, individuals’ lives, and mental health. Evidence for COVID-19 related mental disorders (*e.g.*, anxiety, sleep disturbance, depression, post-traumatic symptoms, and cognitive impairment) is emerging [4]. He *et al.* [5] suggested that COVID-19 might cause psychiatric symptoms in diversified people (*i.e.*, working women, university students, health professionals, and the public). A descriptive study reported that most frontline nurses during COVID-19 pandemic suffered psychological pressures [6].

Temporomandibular joint disorders (TMD) is not a single disease, rather an umbrella term covering 40 diseases related to the disorders of temporomandibular joint, masticatory muscle, and surrounding structures. It can be classified into the second-most common skeletal-muscular disease. Approximately 5%–12% of the US population suffer from TMD, with an economic burden of $4 billion on health system per year [7]. Patients with TMD-related symptoms have no significant sign on imaging examination, while the condylar process has obvious imaging manifestations (*e.g.*, cortical bone resorption and osteophytes) and no related clinical symptoms are detected in patients. Hence, TMD are called “a strange disease” by many dental specialists, of which accurate diagnosis is a challenge for oral general practitioners. The common features of TMD are pain in preauricular area, limited jaw movement, and crackle during opening and closing mouth [8, 9]. The common cause of head and orofacial pain can be attributed to TMD (Head and orofacial pain including painful TMD, toothache, atypical migraine, cluster headache and trigeminal neuralgia) [10]. Sometimes we often encounter patients with TMD referred from the department of neurology in clinical practice. How to diagnose TMD for the first time still remains a challenge for young doctors. Diagnostic Criteria for TMD (DC/TMD) is used for its evaluation, which is made of Axis-I and Axis-II. Axis-I refers to the physical examination of TMD regarding muscle and joint, while Axis-II assesses the psychological status and painful degree of the patients. The new dual-axis of DC/TMD will provide evidence-based standards for clinicians and dentists to evaluate the patients [11, 12]. Up to 15% adults and 7% teenagers suffer TMD chronic pain according to the statistical analysis, which is the main reason for medical treatment. TMD etiology is associated with psychological disabilities, occlusal relationship disorder, and wound and impaired health. TMD-related symptoms can affect the patients’ life quality, daily activity and psychosocial ability [13]. More focus is placed on studies regarding mental health leading to TMD because of the fast-paced contemporary life. Xiang *et al*. [14] conducted a two-sample mendelian study to provide evidence for the major depressive disorder related to TMD’s high risk. A systematic review by Santos *et al*. [15] suggested association between TMD and anxiety.

Patients are often encountered asking if TMD occurs after being infected with COVID-19. As the mental health is associated with these two diseases, is there a relationship of COVID-19 with TMD? A Polish study during COVID-19 pandemic era found that ~50% respondents feared no access to dental services and >50% were afraid of the rising costs, which brought anxiety and stress in dental visit [16]. A previous retrospective study demonstrated that COVID-19 influenced the TMD prevalence [17]. Hannaneh *et al*. [18] conducted cross-sectional research to indicate that TMD symptoms were mostly alleviated in vaccinated people. An Italian survey data suggested that TMD pain was increased after the first year of COVID-19 pandemic [19]. The current evidence indicated association between COVID-19 and TMD, however the causality correlation between the two was lacking. Observational studies confirmed the correlation between TMD and COVID-19, wherein reverse causal relationship and other confounders could not be analyzed.

Mendelian randomization (MR) analysis is an epidemiological method to estimate causal correlation between exposures and outcomes by using SNP as the instrumental variants. MR is not affected by the confounders and reverse causation. It is performed according to the principle of alleles’ random allocation. MR is considered as the “randomized controlled trial in nature” [20]. Relationship between the two diseases is rarely found in MR analyses. Randomized controlled trials thus do not confirm the underlying mechanism. Objectives of this MR are: (1) To verify previous observational studies through new methods; and (2) To resolve the common clinical questions from patients.

## 2. Methods

### 2.1 Study design

A bidirectional two-sample MR using genome-wide association study (GWAS) data was performed to explore causal association between TMD and critically ill-, hospitalized- and reported COVID-19 cases (Figs. 1,2). This MR was designed based on three assumptions: (1) Genetic instrumental variants (IVs) were correlated with the exposure factors (*p *< 5 × 10^−8^/5 × 10^−6^); (2) IVs were independent of the confounders from exposures and outcomes; and (3) IVs had no other path to affect the outcomes [21]. TMD phenotypes GWAS data were available in UK Biobank (UKB). FinnGen was used to validate the difference between European population. COVID-19 phenotypes GWAS statistics from COVID-19 host genetics initiative (HGI) were collected. The results from critically ill-, hospitalized- and reported infected COVID-19 cases were extracted based on the COVID-19-hg GWAS meta-analyses Round 5. Study was conducted by following the STROBE-MR guidelines. The ethical statement was not applicable because of open databases.

**Fig. 1.** The blueprint of bidirectional two-sample MR. Red arrows indicate MR analysis exploring positive causality relationship between TMD and COVID-19; Blue arrows indicate MR analysis exploring reverse causality relationship between TMD and COVID-19; Black arrows indicate common pathway; “×” indicates no relationship. COVID-19: coronavirus disease 2019; UKB: UK Biobank; SNP: Single-nucleotide polymorphism; TMD: temporomandibular joint disorders; HGI: host genetics initiative.

**Fig. 2.** MR analysis flowchart for estimating the causality between COVID-19 and TMD. Red arrows indicate MR analysis at test stage; Blue arrows indicate MR analysis at validation stage; Black arrows indicate co-pathway at test and validation stage. TMD: temporomandibular joint disorders; GWAS: genome-wide association study; UKB: UK Biobank; COVID-19: coronavirus disease 2019; HGI: host genetics initiative; MR: mendelian randomization.

### 2.2 Data source

COVID-19 phenotypes’ GWAS data were obtained from COVID-19-hg GWAS meta-analyses Round 5 GRCh 38 [22]. Severe respiratory confirmed COVID (critically ill COVID-19), hospitalized COVID (hospitalized COVID-19), and the reported severe acute respiratory syndrome coronavirus 2 (SARS-CoV-2) infection (reported infected COVID-19) were included. A total of 3,965,568 Europeans were enrolled in this study. GWAS meta-analyses were based on 14 databases wherein the phenotype of critically ill COVID-19 cases was compared as the diseased cases (N = 4792) with those of population controls (N = 1,054,664). Outcome of hospitalized COVID-19 was based on the patients hospitalized with COVID-19 related symptoms (N = 8316), and the normal population (N = 1,549,095). GWAS data for the hospitalized COVID-19 were analyzed from 21 consortia. The outcomes of population with self-reported COVID-19 infection cases from 35 databases (N = 32,494) were compared with those of controls (N = 1,316,207). TMD GWAS data from UKB (PheCode 526.41) included 217 European ancestry cases, and 456,131 European ancestry controls [23]. Total of 5668 cases and 205,355 controls were extracted from FinnGen R9 related to TMD (The International Classification of Diseases (ICD)-10 version 2016: K07.6) for verifying the difference of European population between two databases [24]. The data source summary of COVID-19 and TMD is provided in Table 1. SNPs associated with the exposure were selected having *p* value < 5 × 10^−8^ for the first correlation assumption. Threshold was lowered to *p* value < 5 × 10^−6^, if <3 SNPs were included. SNPs in strong linkage disequilibrium were removed by setting *r*^2^ = 0.001 and 10,000 kb range. *F* value > 10 was considered strong for evaluating IVs strength as per the following formula [25, 26]:

$$F=\frac{R^{2}\;\times \;\left(N-2\right)}{\left(1-R^{2}\right)}$$

Here, *R*^2^ is the proportion of variance of exposure factor as explained by each instrument, and N the sample size of exposure factor GWAS.

$$R^{2}\;=\;2\times EAF\times \left(1-EAF\right)\times {\;\text{ beta}}^{2}\;\left(SNPs<10\right)$$

$$R^{2}=\frac{2\;\times \;\text{EAF}\;\times \;\left(1\;-\;\text{EAF}\right)\;\times \;\;{\text{beta}}^{2}\;}{\left[\left(2\;\times \;\text{EAF}\;\times \;\left(1\;-\;\text{EAF}\right)\;\times \;\;{\text{beta}}^{2}\right)\;+\;2\;\times \;\text{EAF}\;\times \;\left(1\;-\;\text{EAF}\right)\;\times \;N\;\times \;\text{SE }\;{\left(\text{beta}\right)}^{2}\right]}\;\left(\text{SNPs}\geq 10\right)$$

Here, EAF is the “effect allele frequency” of exposure phenotype, beta the estimated genetic effect on exposure phenotype, and SE (beta) the standard error of genetic effect. The selected SNPs were scanned on PhenoScanner V2 (http://www.phenoscanner.medschl.cam.ac.uk/) based on parameters: *p* = 5 × 10^−8^, *r*^2^ = 0.8, and Build = 37, to eliminate SNPs associated with confounders. *p*-value of SNPs for outcome phenotype was > 5 × 10^−8^/5 × 10^−6^ for the exclusive assumption.

**Table 1.** COVID-19 and TMD data sources.

Traits	Consortium	Cases	Controls	Ancestry
Critically ill COVID-19	14 consortia excluding UKB	4792	1,054,664	European
Hospitalized COVID-19	21 consortia excluding UKB	8316	1,549,095	European
Reported infected COVID-19	35 consortia excluding UKB	32,494	1,316,207	European
TMD	UKB	217	456,131	European
TMD	FinnGen	5668	205,355	European

*COVID-19: the coronavirus disease 2019; TMD: temporomandibular joint disorders; UKB: UK Biobank, a genetic database from United Kingdom; FinnGen: a genetic database from Finland.*

### 2.3 Statistical analyses

Three statistical methods were employed in this study to estimate the potential casual effects between COVID-19 and TMD. Inverse variance weighted (IVW) method was the principal model for MR, wherein intercept term was not considered in the regression and inverse of outcome variance (SE^2^) was used as the weight to fit [27]. The random effects model of IVW was utilized at high heterogeneity, otherwise the fixed effects model of IVW was applied [28]. These SNPs were not pleiotropic in the IVW method. The relationship between outcome and exposure was thus directly proportional. MR-Egger regression was employed for the pleiotropy test to detect pleiotropy effect from SNPs. The weighted median (WM) was applied with the condition that at least 50% of the weight in analysis stemmed from valid IVs [29]. Two-tailed statistical tests were also performed. *p* value was corrected using Bonferroni (I-error/test number, *p *< 0.05/12 ≈ 0.004) if the estimated results from MR analyses were inconsistent.

### 2.4 Sensitivity analyses

The generalized sensitivity analyses included: (1) Gene pleiotropy test; (2) Heterogeneity test; and (3) Leave-one-out method. MR-PROSSO global test and MR-Egger intercept local test were used to detect gene pleiotropy. Results from MR analyses were considered unstable at *p* value < 0.004 and intercept of 0 [30]. Cochran’s Q test was precise to explore the substantial heterogeneity. The leave-one-out method eliminated each SNP one by one, and the meta effect of remaining SNPs was performed. It was observed whether the result changed after each SNP removal. SNP existence had greater impact if result changed greatly. MR analyses results were cautiously explained. MR analyses were conducted by the package TwoSample MR 0.5.7 and MRPRESSO 1.0 in R studio 4.3.1.

### 2.5 Statistical power and sample overlapcalculation

An additional statistical power was used to estimate whether the current sample size was sufficient, in the case where pooled results from MR were negative. Power calculations on MR online calculator (https://sb452.shinyapps.io/power/) explored the statistical power based on: (1) total sample (outcome); (2) cases to controls ratio = 1:X; (3) *R*^2^; (4) estimated odds ratio (OR) for MR; and (5) significance level = 0.004. The current sample size was insufficient and larger sample from GWAS on this subject was required if statistical power was <0.8. The overlap rate was calculated by overlap sample/larger sample, if the sample overlap between two databases was found.

## 3. Results

### 3.1 MR analyses for COVID-19 on TMD (UKB)

No overlap sample size was found between the three COVID-19 phenotypes and TMD in UKB. No evidence of TMD caused by critically ill COVID-19 was detected by selecting 7 SNPs (rs35081325, rs111837807, rs622568, rs10735079, rs77534576, rs2109069 and rs2834163). The selected SNPs were related to exposure phenotype (*F* = 69.81, *R*^2^ = 6.59 × 10^−5^). No confounders were detected. IVW (OR = 0.84, 95% confidence interval (CI) = 0.60–1.17, *p *= 0.295) method showed that TMD was not related to critically ill COVID-19. MR-Egger (OR = 1.11, 95% CI = 0.46–2.67, *p *= 0.818) and WM (OR = 0.81, 95% CI = 0.55–1.21, *p *= 0.308) methods depicted consistent results (Fig. 3). Leave-one-out sensitivity test demonstrated robust result (Fig. 4A). MR-PROSSO global test (*p *= 0.976) and MR Egger intercept local test (intercept = −0.091, *p *= 0.517) revealed no gene pleiotropy. Cochran’s Q test (Q = 1.406, *p *= 0.965) did not detect substantial heterogeneity (Table 2). Statistical power calculations indicated insufficient sample size (power = 0.2%). For the hospitalized COVID-19 risk factor, 7 SNPs (rs41264915, rs35081325, rs111837807, rs622568, rs1859330, rs2109069, rs13050728) with strong correlation (*F* = 73.84, *R*^2^ = 4.74 × 10^−5^) were analyzed to estimate the genetic causal relationship between hospitalized COVID-19 and TMD. IVW (OR = 0.78, 95% CI = 0.51–1.19, *p *= 0.246) showed no statistical difference like those of MR-Egger (OR = 0.96, 95% CI = 0.34–2.69, *p *= 0.940) and WM (OR = 0.78, 95% CI = 0.47–1.31, *p *= 0.349) (Fig. 3). No heterogeneity was detected (Q = 1.174, *p *= 0.978). No evidence of gene pleiotropy was found in MR analyses (MRPRESSO global test *p *= 0.979, MR-Egger intercept = −0.016,* p *= 0.928) (Table 2). MR analyses results were robust as achieved from the leave-one-out method (Fig. 4B). However, the current exposure sample size was insufficient (power = 0.2%). No evidence existed in MR analyses for the reported infected COVID-19 affecting TMD (IVW: OR = 0.78, 95% CI = 0.23–2.67, *p *= 0.698; MR-Egger: OR = 0.92, 95% CI = 0.03–24.71, *p *= 0.965; WM: OR = 0.61, 95% CI = 0.15–2.55, *p *= 0.499) (Fig. 3). Heterogeneity test, gene pleiotropy test, and leave-one-out method demonstrated stable results (Table 2; Fig. 4C). Four SNPs (rs35508621, rs579459, rs1859330, rs2109069) had strong correlation with reported infected COVID-19 (*F* = 51.48, *R*^2^ = 3.82 × 10^−5^). Statistical power calculation reflected insufficient sample size (power = 0.2%).

**Fig. 3.** A forest summary plot for COVID-19 on TMD (UKB). TMD: temporomandibular joint disorders; UKB: UK Biobank; SNP: Single-nucleotide polymorphism; COVID-19: the coronavirus disease 2019; WM: Weighted median; IVW: Inverse variance weighted; MR: Mendelian randomization; OR: Odds ratio; CI: Confidence interval; *: No practical meaning.

**Table 2.** Sensitivity analyses for COVID-19 and TMD.

Exposure	Outcome	No. of SNPs	MR-Egger regression		MR-PRESSO	Heterogeneity test	
			Intercept	*p* Intercept	*p*	Cochran Q	*p*
Critically ill COVID-19	TMD in UKB	7	−0.091	0.517	0.976	1.406	0.965
Hospitalized COVID-19	TMD in UKB	7	−0.016	0.928	0.979	1.174	0.978
Reported infected COVID-19	TMD in UKB	4	−0.050	0.687	0.625	2.007	0.571
TMD in UKB	Critically ill COVID-19	7	−0.038	0.420	0.303	7.133	0.309
TMD in UKB	Hospitalized COVID-19	6	−0.014	0.704	0.416	5.122	0.401
TMD in UKB	Reported infected COVID-19	7	−0.019	0.276	0.498	5.579	0.472
Critically ill COVID-19	TMD in FinnGen	7	0.009	0.707	0.607	5.023	0.541
Hospitalized COVID-19	TMD in FinnGen	6	−0.001	0.969	0.362	6.703	0.244
Reported infected COVID-19	TMD in FinnGen	4	−0.014	0.679	0.772	1.255	0.740
TMD in FinnGen	Critically ill COVID-19	14	0.006	0.808	0.672	10.800	0.627
TMD in FinnGen	Hospitalized COVID-19	13	0.019	0.373	0.807	7.194	0.845
TMD in FinnGen	Reported infected COVID-19	14	0.001	0.952	0.576	12.022	0.526

*COVID-19: the coronavirus disease 2019; TMD: temporomandibular joint disorders; UKB: UK Biobank, a genetic database from United Kingdom; FinnGen: a genetic database from Finland; SNPs: single-nucleotide polymorphisms, a mutation in DNA sequence caused by a change in a single nucleotide-A, G, C and T; MR: Mendelian randomization.*

**Fig. 4.** A leave-one-out plot for COVID-19 on TMD (UKB). (A) Critically ill COVID-19 on TMD (UKB); (B) Hospitalized COVID-19 on TMD (UKB); and (C) Reported infected COVID-19 on TMD (UKB). MR: Mendelian randomization.

### 3.2 MR analyses for TMD (UKB) on COVID-19

Reverse MR was conducted to explore reverse causality between TMD and COVID-19. Fig. 5 exhibits MR results of three COVID-19 phenotypes. No significant evidence was found for COVID-19 being affected by TMD. IVW results (OR = 1.01, 95% CI = 0.97–1.05, *p *= 0.795) revealed that TMD was not associated with critically ill COVID-19. MR-Egger, and WM demonstrated consistent results. Seven SNPs (*F* = 22.63, *R*^2^ = 4.96 × 10^−5^) were selected for the MR analyses. Pleiotropy test, heterogeneity test, and leave-one-out test suggested robust results (Table 2; Fig. 6A). However, the statistical power was only 0.2%. IVW results (OR = 0.98, 95% CI = 0.95–1.01, *p *= 0.185) showed that TMD was not related to hospitalized COVID-19. These results were like those of MR-Egger and WM (Fig. 5). Six SNPs (*F* = 22.84, *R*^2^ = 5.00 × 10^−5^) were associated with TMD. Sensitivity analyses (leave-one-out, MR-Egger intercept, Cochran’s Q test, and MR-PRESSO) indicated stable results (Table 2; Fig. 6B). Statistical power of MR was 0.2%. IVW results (OR = 1.01, 95% CI = 0.99–1.03, *p *= 0.246) indicated that TMD did not affect reported infected COVID-19. MR-Egger and WM validated the results (Fig. 5). Seven selected SNPs (rs112467061, rs11577938, rs117436048, rs149752386, rs150764461, rs75876538, rs78579250) had strong correlation (*F* = 22.63, *R*^2^ = 4.96 × 10^−5^), which was same as with critically ill COVID-19 phenotype. Sensitivity analyses demonstrated robust results (Table 2; Fig. 6C). The sample size was insufficient for MR analysis (power = 0.2%).

**Fig. 5.** A forest summary plot for TMD (UKB) on COVID-19. TMD: temporomandibular joint disorders; UKB: UK Biobank; SNP: Single-nucleotide polymorphism; COVID-19: the coronavirus disease 2019; WM: Weighted median; IVW: Inverse variance weighted; MR: Mendelian randomization; OR: Odds ratio; CI: Confidence interval; *: No practical meaning.

**Fig. 6.** A leave-one-out plot for TMD (UKB) on COVID-19. (A) TMD (UKB) on critically ill COVID-19; (B) TMD (UKB) on hospitalized COVID-19; and (C) TMD (UKB) on reported infected COVID-19. MR: Mendelian randomization.

### 3.3 MR analyses for COVID-19 on TMD (FinnGen)

MR analyses were repeated for COVID-19 on TMD in FinnGen consortium. Overlap size rate between the critically ill COVID-19 on TMD was 19.92% in FinnGen. Seven SNPs (rs10735079, rs111837807, rs2109069, rs2237698, rs2834163, rs35081325, rs77534576) with strong correlation (*F* = 68.54, *R*^2^ = 6.47 × 10^−5^) were included in MR analyses. No evidence was found for the causal relationship between COVID-19 and TMD by IVW (OR = 1.05, 95% CI = 0.99–1.12, *p *= 0.123), ME-Egger (OR = 1.02, 95% CI = 0.87–1.20, *p *= 0.826), and WM (OR = 1.05, 95% CI = 0.96–1.14, *p *= 0.289) (Fig. 7). Four sensitivity analyses depicted no bias in the MR (Table 2; Fig. 8A). Statistical power calculation was 0.2% for the sample size. IVW (OR = 1.06, 95% CI = 0.96–1.17, *p *= 0.228), MR-Egger (OR = 1.07, 95% CI = 0.83–1.37, *p *= 0.646), and WM (OR = 1.06, 95% CI = 0.95–1.18, *p *= 0.271) exhibited no statistical difference using 6 SNPs (*F* = 80.41, *R*^2^ = 7.59 × 10^−5^), for hospitalized COVID-19 on TMD (Fig. 7). The overlap sample size rate of 13.55% was found between two consortia. No bias was found in four sensitivity analyses (Table 2; Fig. 8B). The sample size (power = 0.2%) was insufficient in TMD (FinnGen). IVW (OR = 1.08, 95% CI = 0.85–1.37, *p *= 0.539) suggested no statistical difference for causal relationship between reported infected COVID-19 and TMD. MR-Egger, and WM showed consistence results (Fig. 7). Four SNPs (rs35508621, rs579459, rs1859330, rs2109069) were included with no weak bias (*F* = 51.48, *R*^2^ = 4.86 × 10^−5^). Sensitivity analyses depicted robust results (Table 2; Fig. 8C) with statistical power of 0.2%. There was 15.65% overlap sample size between reported infected COVID-19 and TMD (FinnGen).

**Fig. 7.** A forest summary plot for COVID-19 on TMD (FinnGen). TMD: temporomandibular joint disorders; UKB: UK Biobank; SNP: Single-nucleotide polymorphism; COVID-19: the coronavirus disease 2019; WM: weighted median; IVW: Inverse variance weighted; MR: mendelian randomization; OR: Odds ratio; CI: Confidence interval; *: No practical meaning.

**Fig. 8.** A leave-one-out plot for COVID-19 on TMD (FinnGen). (A) Critically ill COVID-19 on TMD (FinnGen); (B) Hospitalized COVID-19 on TMD (FinnGen); and (C) Reported infected COVID-19 on TMD (FinnGen). MR: Mendelian randomization.

### 3.4 MR analyses for TMD (FinnGen) on COVID-19

The reverse relationship between two diseases was validated. Fig. 9 summarizes MR results of causal association between TMD and COVID-19. IVW (OR = 0.98, 95% CI = 0.87–1.11, *p *= 0.760), MR-Egger (OR = 0.96, 95% CI = 0.78–1.18, *p *= 0.709), and WM (OR = 0.97, 95% CI = 0.82–1.14, *p *= 0.689) showed no evidence of critically ill COVID-19 being affected by TMD. Fourteen SNPs (rs10882591, rs111843207, rs114568702, rs12466258, rs138516336, rs16995253, rs2058908, rs6752506, rs67939396, rs72935077, rs76537824, rs77640278, rs78882783, rs903554) were extracted with strong IVs (*F* = 22.63, *R*^2^ = 9.48 × 10^−5^). Cochrane’s Q test did not detect substantial heterogeneity (Q = 10.800, *p *= 0.627). MR-PRESSO global test (*p *= 0.672), and MR-Egger regression intercept test (intercept = 0.006, *p *= 0.808) suggested no gene pleiotropy (Table 2). The leave-one-out method eliminated selected SNPs one by one, and no bias was detected in this MR (Fig. 10A). Statistical underperformance was found in outcome sample (power = 0.2%). IVW (OR = 0.97, 95% CI = 0.89–1.07, *p *= 0.567), MR-Egger (OR = 0.92, 95% CI = 0.78–1.07, *p *= 0.301), and WM (OR = 0.96, 95% CI = 0.84–1.09, *p *= 0.503) results had no evidence of hospitalized COVID-19 being associated with TMD, *via* the 13 TMD-related SNPs (*F* = 22.56, *R*^2^ = 9.45 × 10^−5^) (Fig. 9). Four sensitivity analyses (*i.e.*, leave-one-out, MR-Egger regression intercept, Cochrane’s Q test, and MR-PRESSO) found no significant bias (Table 2; Fig. 10B). The outcome sample size was insufficient because of 0.2% statistical power. A total of 14 SNPs were selected for reported infected COVID-19, which were same as the critically ill COVID-19 phenotype. IVW (OR = 0.99, 95% CI = 0.95–1.04, *p *= 0.730) had same results as those of MR-Egger (OR = 0.99, 95% CI = 0.91–1.07, *p *= 0.805), and WM (OR = 0.99, 95% CI = 0.93–1.06, *p *= 0.826) (Fig. 9). No biases were detected in Cochrane’s Q test (Q = 12.022, *p *= 0.526), MR-PRESSO (*p *= 0.576), and MR-Egger regression intercept test (intercept = 0.001, *p *= 0.952) (Table 2). The leave-one-out test showed robust results (Fig. 10C). The statistical power of TMD on reported infected COVID-19 was 0.2%.

**Fig. 9.** A forest summary plot for TMD (FinnGen) on COVID-19. TMD: temporomandibular joint disorders; UKB: UK Biobank; SNP: Single-nucleotide polymorphism; COVID-19: the coronavirus disease 2019; WM: weighted median; IVW: Inverse variance weighted; MR: mendelian randomization; OR: Odds ratio; CI: Confidence interval; *: No practical meaning.

**Fig. 10.** A leave-one-out plot for TMD (FinnGen) on COVID-19. (A) TMD (FinnGen) on critically ill COVID-19; (B) TMD (FinnGen) on hospitalized COVID-19; and (C) TMD (FinnGen) on reported infected COVID-19. MR: Mendelian randomization.

## 4. Discussion

A bidirectional two-sample MR based on HGI, UKB and FinnGen was conducted in this study. TMD-related GWAS data from UKB was employed as the test set to explore bidirectional correlation between the two sides. FinnGen as a validation set further confirmed the results. MR analyses results revealed no significant causal correlation between COVID-19 and TMD. MR-Egger intercept, and MR-PRESSO tests indicated no other confounders affecting IVs, which were consistent with the results from PhenoScanner V2.

Once the COVID-19 was subsided, a study reported that the patients diagnosed with painful TMD were 3.3 times higher compared to pre-pandemic period. It suggested that COVID-19 had no association with TMD. This conclusion was consistent with the MR analyses of this study. It was also found therein that females were more prone to be affected by TMD than males during COVID-19 pandemic [31]. It was inferred that sex hormones mediated increased susceptibility to TMD in women infected with COVID-19 [32]. Another retrospective study confirmed the results of these MR analyses. Yap *et al*. [33] considered that COVID-19 had no adverse impact on pain-related TMD or intra-articular TMD based on Chinese and Korean patients. Sex and age were more important in TMD progression. However, all the GWAS data in our MR analyses was derived from mixed European population instead of East Asian ancestry. More TMD related GWAS consortium (*e.g.*, BioBank, Japan) on East Asian ancestry could be employed in future for further related studies.

A systematic review from Italy reported an association between COVID-19 and the increased TMD incidence, which was contrary to the conclusion of this study [34]. COVID-19 itself was unable to cause TMD. Related reports informed about more people having TMD because of job loss, and lockdowns during COVID-19 pandemic. Four articles were included in that systematic review based on the current evidence. It was speculated herein that confounders in the systematic review would have impact on results interpretation. TMD as the generic term was discussed in this study. There might be a positive effect in some sub-TMD, while in others could have negative effect, and the total effect showed no impact. A prospective study from Moath *et al*. [35] suggested that wearing a face mask during COVID-19 had an influence on TMD. Individuals thus used appropriate mask size for the face shape. The jaw movements could lead to TMD because people tend to push the jaw forward while wearing a face mask. It was considered herein that TMD prevalence might decrease if face mask was designed to restrict the jaw-forward movement. Literature revealed that the harmful factors from work environment were closely related to TMD development [36]. For instance, the long-term exposure of dental turbine noise might induce hearing problems, nerve system irritation, and fatigue in dentists to ultimately develop TMD. It was speculated in this study that the occupation might have role in TMD prevalence.

Statistical result in this study was cautiously explained. Because of the negative results, statistical power was calculated to estimate whether sample size was sufficient. The statistical power of all MRs was 0.2%, and there might be false negative in MR. MR-Egger (reported infected COVID-19 on TMD in UKB) was characterized by wide confidence interval (95% CI: 0.03–24.71), which indicated insufficient sample size. The demands were thus high for more GWAS data related to COVID-19 and TMD. There was an overlap sample size between HGI and FinnGen (13.55%~19.92%). Burgess *et al*. [37] considered that the bias results were uncertain when using GWAS consortium with partially overlap sets of population. The overlap sample in two-sample MR could increase type-I error. Two samples tend to be single with increase in overlapping samples. Bayesian MR statistical models had been developed which addressed the sample overlap issue and achieved similar accuracy as that of traditional MR. They would further be applied in medicine field in future [38].

From psychology perspective, an attempt was made in this study to explain TMD occurring after the infected COVID-19. Three personality types related to the evolution of somatic diseases were: Type A (coronary), Type C (cancer-prone), and Type D (distressed) [39]. A quantitative analysis from Magdalena *et al*. [40] demonstrated that type D personality might be associated with the development of stomatognathic system disorders. The principle was based on response level of cortico-reticular loop determining the activation level. Zach *et al*. [41] from the psychological study reported significant difference in somatization score between TMD and non-TMD individuals. For instance, a patient with psychological problems would consult whether TMD-related symptom was the result of COVID-19. It could be possible that the patient was affected by non-pain TMD symptoms before infected COVID-19. Home isolation during COVID-19 pandemic aggravated TMD procession towards pain symptoms as TMD itself was a bio-socio-psychological-factor [42]. An epidemiological study from Emodi-Perlman *et al.* [43] depicted that COVID-19 had adverse impact on psychological status of Israeli and Polish populations, which exaggerated the bruxism and TMD symptoms. An observational study suggested that higher psychological distress, depressive symptoms, and anxiety were found in individuals with orofacial pain (including pain TMD) during COVID-19 pandemic [44]. This study also confirmed that the patients infected with COVID-19 had no worries of SARS-CoV-2 causing TMD. On the contrary, excessive psychological stress (*i.e.*, hysteria, anxiety, and depression) during pandemic would have adverse impact on temporomandibular joint, which was consistent with the outcomes of other studies.

There were certain limitations in this study. The related-TMD summary data in GWAS consortium were the pooled results of European ancestry (*e.g.*, TMD in this study was not accurately classified into pain TMD and non-pain TMD, and the population could be stratified into men, women, elderly, and young in future). SNPs selected as IVs might not be strongly associated with TMD (*p *< 5 × 10^−6^) because of weak statistical power of MR on this topic.

## 5. Conclusions

It is concluded that there is no causal correlation between the two diseases. COVID-19 itself does not have impact on TMD, and TMD does not increase the susceptibility to COVID-19. Patients with TMD-related symptoms should not feel threatened by COVID-19. During COVID-19 pandemic, excessive psychological stress caused by COVID-19 might act as a mediator between the two diseases [45]. Both general practitioners and dental specialists should treat patients in accordance with biopsychosocial model, in which patients’ personality, emotion, living environment and social relationship should be taken into account [46]. In addition, Previous observational studies on two diseases were not be validated further due to insufficient statistical power of this study. Relationship between the two sides needs verification through more external studies in the future.
